# Costly Prosociality, Familiarity Among Peers, and School Climate: A Predictive Model

**DOI:** 10.3390/bs16101754

**Published:** 2026-09-26

**Authors:** David Cuadra-Martínez, Rodrigo Landabur, José Sandoval-Díaz, Daniel Pérez-Zapata

**Affiliations:** 1 Departamento de Psicología, Universidad de Atacama, Copiapó 1531772, Chile; david.cuadra@uda.cl; 2 Centro de Estudios Ñuble, Universidad del Bio-Bio, Concepción 4081112, Chile; jsandoval@ubiobio.cl; 3 School of Psychology, University of Birmingham, Birmingham B15 2TT, UK; d.perezzapata@bham.ac.uk

**Keywords:** costly prosociality, beneficiary familiarity, school climate

## Abstract

The objective of this study is to examine the associations between costly prosociality toward known and unknown school peers and well-being, school aggression, and school climate among Chilean secondary school students by estimating structural paths from costly prosociality to these variables. A correlational and explanatory quantitative study was conducted using a sample of 529 Chilean secondary school students recruited through convenience sampling. Likert-type self-report scales were administered to measure costly prosociality, school aggression, well-being, and school climate. HCPB toward both known and unknown peers was positively associated with well-being and school climate. In the structural models, the model involving known peers showed a significant negative path to school aggression, whereas the model involving unknown peers did not show a significant path to school aggression. The two structural models showed different descriptive patterns of associations, with the model involving unknown peers accounting for 34% of the variance in school climate, while the model involving known peers accounted for 27%. These findings provide descriptive evidence of distinct patterns of associations between costly prosociality and school-related variables according to beneficiary familiarity. In the discussion section, results are analyzed in light of the literature.

## 1. Introduction

Prosociality is fundamental to people’s development, social cohesion, and the progress of societies (M. Kim & Sul, 2026). In schools, it has been linked to reduced violence (Manzano-Sánchez et al., 2021), increased well-being (Li et al., 2023), and improved school climate (C. Wang et al., 2022), three dimensions that are prioritized in school policy worldwide (Țîroiu, 2023). However, less is known about how prosociality and these school variables interact when help entails major costs to the benefactor (Hammond et al., 2023).

Prosociality encompasses a broad range of voluntary behaviors intended to benefit others, which may vary considerably in the degree of cost or risk assumed by the benefactor. Costly prosociality represents a specific form of prosocial behavior in which helping involves substantial cognitive, social, emotional, or material costs or risks for the benefactor.

Across the multiple expressions of prosociality, the cost of help is a key factor in the decision to deploy it (Wu et al., 2026). Despite its importance, however, more research is needed to gain a clearer understanding of its modes of expression (Armstrong-Carter & Telzer, 2025). This need is especially pressing in school settings, where students can promote violence, be passive observers, or behave prosocially to defend victims (Bautista et al., 2024).

Costly prosociality is a type of help that carries major sacrifices and risks for the benefactor. Specifically, it differs from other types of prosociality due to its high cognitive, social, and material cost (Padilla-Walker et al., 2018) and because of how fundamental it can be to the beneficiary (Binder et al., 2026).

Nowadays, bodies such as the United Nations Educational, Scientific and Cultural Organization (UNESCO, 2024) regard students as active agents in efforts to improve school climate and reduce school violence. In Latin America, several educational policies and school climate programs have incorporated bystanders or active observers as a way of addressing school violence. Although limited evidence is available regarding these types of interventions, the systematic review conducted by Rodas et al. (2025) shows that bullying reduction programs have been implemented in several educational institutions, strengthening social skills, peer support, and prosocial behaviors.

Precisely, high-cost prosocial behavior (HCPB) comprises actions such as defending the victims of physical violence (Nielson et al., 2017) and socially including persons who have been excluded (Padilla-Walker et al., 2018). Adolescence is an especially critical stage regarding the manifestation of these behaviors. According to Armstrong-Carter and Telzer (2025), young people can lie and/or break social norms to protect relatives, friends, and unknown people, even when this entails personal risk. It is worth noting, though, that this behavior can differ in connection with the benefactor’s developmental stage, sex, and degree of familiarity with the beneficiary (Armstrong-Carter & Telzer, 2025).

The evidence presented indicates that it is necessary to gain a clearer understanding of the multiple expressions of prosociality at school, especially when it entails costs and risks for the benefactor. It is relevant to understand how HCPB interacts with school climate and school violence, as students may misinterpret the victims’ need for help and adopt controversial measures to defend them (Bruno et al., 2020). Furthermore, it is worth noting that the association between HCPB and well-being has not been conclusively determined (Hirani et al., 2022; Tomar, 2023). Students who engage in HCPB are more likely to intervene in order to stop school violence; thus, they actively contribute toward a positive school climate (Kohen et al., 2019). However, the constant risk and stress involved might result in burnout.

In consequence, examining costly prosociality toward known and unknown peers, considering its variation by sex and grade, constitutes a pertinent approach aimed at describing the patterns of association with school climate, aggression among peers, and student well-being. These variables have been reported to be essential components of school functioning (UNESCO, 2024) and are addressed in the present study.

### 1.1. High-Cost Prosocial Behavior

Prosociality is a behavior that consists of voluntarily helping others to solve their needs and difficulties (Binder et al., 2026). When it entails major risks or sacrifices for the benefactor, it constitutes a high-cost prosocial behavior (HCPB), that is, a type of helping behavior associated with large resource investments, more effort, and greater cognitive, emotional, and behavioral risks/costs (Padilla-Walker et al., 2018). This manifestation of prosociality has not been extensively researched, especially in educational settings (Hammond et al., 2023).

Armstrong-Carter and Telzer (2025) identified several actions as expressions of risky prosociality, including lying, defending, losing personal benefits or reputation, and breaking rules to help someone. Similarly, Padilla-Walker et al. (2018) have linked risky prosociality with defending and including others. For example, in schools, it is exhibited by students who actively stop a fight or invite a classmate who has been rejected by their peers to join them in a school activity.

Research has shown that HCPB is a sign of greater community commitment (Gini et al., 2011), being linked to increased self-efficacy, personal growth (Tomar, 2023), and moral development (Gini et al., 2011). Armstrong-Carter et al. (2021) state that adolescents must meet their social connection needs, which may cause them to seek socially gratifying but risky experiences that involve helping others. In addition, traits such as higher tolerance of uncertainty, sensation seeking, and greater concern over social reputation have been linked to higher levels of risky prosociality in young people (Armstrong-Carter & Telzer, 2025).

At school, students’ interactions allow this behavior to develop, given the opportunities available for practicing various modes of coexistence with peers, which increases the complexity of prosocial behaviors (Hammond & Brownell, 2015).

Some authors have found differences in HCPB associated with age and sex, but the available evidence is insufficient and thus inconclusive. Regarding age, Hammond and Brownell (2015) have specified that prosocial behavior is less prevalent in early adolescence, and that it increases with age due to a stronger need for social integration. With respect to sex, in childhood, girls tend to exhibit more prosocial behaviors aimed at comforting others, while boys engage in riskier prosocial behaviors (Hammond & Brownell, 2015). Nevertheless, Nielson et al. (2017) assert that these disparities are due to different ways of behaving prosocially as a result of gender stereotypes, whose influence is stronger during adolescence. Thus, gender stereotypes may shape the expression of HCPB by encouraging male adolescents to engage in defensive prosocial behaviors toward others, whereas female adolescents may be more strongly encouraged to express prosociality through kindness and compliance (Nielson et al., 2017).

### 1.2. Costly Prosociality and Beneficiary Familiarity

Evidence suggests that high-cost prosocial behavior may vary depending on the relationship with the beneficiary. For example, Monks and Rix (2024) found greater defending behavior among friends during childhood. Similarly, Armstrong-Carter et al. (2021) found that prosocial behavior was related to adolescents’ motivation for social connection, whereas Padilla-Walker et al. (2018) showed that prosocial behavior varies depending on whether it is directed toward family members, friends, or strangers. In educational contexts, recent studies suggest that peer relationships may also contribute to this variation: during the transition to secondary education, social dominance has been associated with riskier defending behaviors toward unknown peers, whereas friendship-based relationships become more salient later, with prosocial behavior tending to be directed toward peers closer to the benefactor (Armstrong-Carter & Telzer, 2025; Waasdorp et al., 2022). Taken together, these findings suggest that familiarity with the beneficiary is a relevant dimension for understanding variation in high-cost prosocial behavior.

### 1.3. Costly Prosociality, School Climate, and School Aggression

School climate is defined as the shared perceptions of students, teachers, and families regarding the relationships, practices, norms, and conditions that characterize the daily life of the school and the experiences of the educational community (Castro et al., 2026).

As for school violence, it is a worldwide problem that represents an urgent challenge for schools (Mosito & Sitoyi, 2024). It is a broad-ranging phenomenon that encompasses multiple types of harm and victimization that take place in educational settings, including physical, psychological, and sexual violence, along with other types of aggression among members of the school community. Its manifestations include school aggression, defined as intentional behaviors of a physical, verbal, or relational nature that occur at school and which are aimed at causing harm or displeasure to others (López & Orpinas, 2012). The present study covers school aggression, since it is considered to be one of the most frequent manifestations of school violence.

With respect to the relevance of school climate, it has been shown to be essential for learning and education (M. T. Wang & Degol, 2016), fostering safe and participatory learning contexts and influencing students’ socioemotional development (Castro et al., 2026). By contrast, school aggression has deep and lasting negative impacts, affecting the mental health, quality of life, well-being, and academic performance of both victims and assailants (İlğan et al., 2026).

Authors have stressed that, to reduce the various expressions of school violence and improve school climate, it is essential to help students to develop socioemotional skills, including problem-solving, empathy, and assertive communication (Cunha et al., 2025).

In this context, prosociality is a key behavior. There is evidence that support among peers improves school climate and reduces violence (Freeman et al., 2024). For instance, Waasdorp et al. (2022) found that HCPB improves two fundamental dimensions of school climate: commitment to the school and perception of the school as a safe space. In addition, when students defend victims of aggression, bonds of friendship are likely to emerge between the benefactor and the beneficiary (Monks & Rix, 2024). Regarding school violence, student witnesses of violence are a major factor in its development: when they back the assailants, bullying is more frequent; in contrast, when they support and defend the victims, attacks on peers are reduced (Xie & Ngai, 2020). This could also result in the establishment of an anti-bullying culture and increased student well-being (Willems et al., 2024), emerging as a protective factor against bullying (Chicote-Beato et al., 2026). However, HCPB within the context of school violence can also be controversial depending on whether students employ adaptive or maladaptive defensive strategies (Lambe & Craig, 2020).

### 1.4. Costly Prosociality and Well-Being

Psychological well-being is defined as a person’s subjective experience of positive psychological states such as pleasure, personal realization, beneficial interpersonal relationships, personal development, and a sense of purpose and control over their life (Dhanabhakyam & Sarath, 2023).

Nowadays, well-being promotion in schools has become a core objective of educational policy worldwide (Hossain et al., 2023). Well-being is not only the basis for students’ successful learning, but also one of the goals of education in this century (Govorova et al., 2020).

Among the causes of well-being, the literature lists biopsychosocial factors such as adverse life events, genetic predispositions, family problems, neurobiological determinants, and school conditions (Storch & Yang, 2024). Among the latter factors, it has been established that exposure to tense and violent school contexts has a negative impact on students’ mental health and psychological well-being (Sibisi et al., 2024).

With respect to prosocial behavior and well-being, the literature has revealed a strong link between these variables, though there is not enough evidence regarding the conditions under which a positive or negative association is produced (Hirani et al., 2022; Tomar, 2023). There is evidence of greater life satisfaction and well-being (Tomar, 2023) in students who engage in HCPB. Nevertheless, authors have also identified a higher prevalence of mental health issues like anxiety and depression in young people who take on the role of protecting others (Greene et al., 2017), feelings of fear and concern due to reprisals stemming from helping others, and higher rates of emotional dysregulation in students who witness violence, including those who defend victims (Fabris et al., 2022). In addition, even though empirical studies have mainly examined how school climate can improve student well-being (Aldridge & McChesney, 2018), the broaden-and-build theory proposes that well-being facilitates the construction of social resources, strengthening interpersonal trust, openness, and relationship quality (Fredrickson, 2001). On an empirical level, a study by E. S. Kim et al. (2024) shows that positive emotions lead to better social functioning and integration over time, suggesting the presence of a plausible mechanism through which well-being could be associated with a more favorable school climate.

The general objective of this study is to examine the associations between costly prosociality toward known and unknown school peers and well-being, school aggression, and school climate among Chilean secondary school students by estimating structural paths from costly prosociality to these variables. On a specific level, the objectives of the study are: (a) to characterize HCPB levels in secondary school students, comparing by sex, grade, and type of beneficiary (known/unknown); (b) to determine the statistical relationship among student HCPB toward known and unknown peers, well-being, school aggression, and school climate; and (c) to generate structural equation models to analyze the relationships of HCPB toward known and unknown peers with psychological well-being, school aggression, and school climate, also considering the structural relationships of psychological well-being and school aggression with school climate.

The following hypotheses are advanced: (a) HCPB differs by type of beneficiary, sex, and grade; (b) HCPB toward known and unknown students is linked to the variables well-being, school aggression, and school climate; (c) HCPB toward known and unknown peers is expected to have statistically significant structural paths to psychological well-being, school aggression, and school climate. In addition, psychological well-being and school aggression are expected to have statistically significant structural paths to school climate.

## 2. Materials and Method

### 2.1. Design

This correlational and explanatory study utilized a cross-sectional design, as the variables were not manipulated and data were collected at a single moment. A quantitative methodology was used. This design was considered appropriate for testing theoretically specified structural relationships among costly prosociality, well-being, school aggression, and school climate, and for estimating the degree of variation in school climate accounted for by the proposed models. Kline (2023) emphasizes that, when specifying structural models, researchers should provide a theoretical rationale for the directionality of the proposed relationships, particularly when the research design does not include formal elements of temporal precedence that support causal inference. In cross-sectional designs, theoretically justified directional relationships may therefore be specified, but their interpretation requires caution regarding causal priority. Accordingly, the structural paths examined in the present study are interpreted as theoretically grounded statistical relationships, and the term “predictive” refers to the statistical estimation of directional paths and the variance accounted for within the structural models rather than to causal prediction.

### 2.2. Participants and Sampling

Convenience sampling—a type of non-probability sampling—was used, resulting in the inclusion of 529 secondary school students from two public schools in the Atacama Region of Chile. Sample size was estimated with the A-priori Sample Size Calculator for Structural Equation Models developed by Soper (2026), considering six latent variables and 40 observed variables, an effect size of 0.20, a significance level of 0.05, and a statistical power of 0.80. The calculator reported a minimum recommended sample size of 403 participants. The final sample comprised 529 students. They were 15.59 years old on average (SD = 1.26), 52% were girls and 48% boys, and 47% attended school 1 and 53% school 2. Both schools were public schools administered by the Atacama Local Public Education Service (SLEP Atacama). School 1 had an enrollment of 1001 students, offered a polyvalent educational modality, and had a Social Vulnerability Index (SVI) of 93%. School 2 had an enrollment of 482 students, also offered a polyvalent educational modality, and had an SVI of 80%.

### 2.3. Information Collection

This study is part of a Fondecyt Regular project (N° 1250553) certified by the Research Ethics Committee (Certificado N° 04-26) of the University of Atacama, located in northern Chile. The study was explained to the students and their parents. The participants were enrolled after completing informed assent and consent procedures. Information collection instruments were administered in the school facilities and during regular school hours.

(a) Sociodemographic survey to collect student characterization data, including sex, age, grade, and school.
(b) Multidimensional Measure of Prosocial Behavior by Nielson et al. (2017). Within the context of the larger project to which this study belongs, the Multidimensional Measure of Prosocial Behavior by Nielson et al. (2017) was culturally adapted and validated for use in Chile. The cultural adaptation involved review by expert judges and a pilot test consisting of 10 cognitive interviews with students to assess the cultural and linguistic appropriateness of the items. It is a self-report scale composed of 20 Likert items aimed at measuring various types of prosocial behavior in adolescents in the United States and Europe. It comprises five dimensions: defending, emotional support, inclusion, physical helping, and sharing. As part of the validation of this scale, a second-order model and two first-order factors of high-cost prosocial behavior were included, using the dimensions defending and inclusion (Nielson et al., 2017). These two dimensions were selected because they specifically capture costly prosocial actions involving defense against school violence and the social inclusion of excluded peers, which are the manifestations of HCPB examined in the present study. Each dimension comprises four Likert-type items (Never = 1; Always = 5) that measure the help provided under conditions of school aggression and exclusion (for example, “I step in to stop fights”; “If a person is new to a group, I make an effort to include that person”). The validation of the second-order factor or costly prosociality exhibited adequate fit indicators (*χ*^2^ = 41.42, df = 17, *p* < 0.001, *CFI* = 0.996, *TLI* = 0.993, *RMSEA* = 0.052, *SRMR* = 0.046) and reliability levels ranging from 0.602 to 0.829 for the overall measure and its two factors. Based on the recommendations by Nielson et al. (2017), to measure HCPB by type of beneficiary—known/unknown student—the participants were shown the 8 items from the defending and inclusion dimensions switching the beneficiary’s status: (a) Condition 1, known beneficiary, “Answer thinking of school peers you know”; (b) Condition 2, unknown beneficiary, “Answer thinking of school peers you DON’T know”. The questionnaire was administered as a booklet, with the known-peer condition presented first. This was followed by the Caprara et al. (2005) prosociality measure, the school aggression measure, the well-being measure, and the CUVE school violence measure. The eight HCPB items were then presented again in the final section of the booklet, referring to unknown peers. Thus, the two HCPB conditions were separated by 63 items from other measures. Peer familiarity was operationalized as whether participants knew or did not know the school peer, without specifying a particular degree of friendship or closeness.

Because the same eight items were administered under both beneficiary conditions, we examined their measurement equivalence across known- and unknown-peer conditions using confirmatory factor analysis with WLSMV estimation for ordinal responses. Separate second-order CFAs showed good model fit in both conditions. For known peers, the model showed *χ*^2^(17) = 22.393, *p* = 0.170, CFI = 0.997, TLI = 0.994, RMSEA = 0.025 (90% CI [0.000, 0.049]), and SRMR = 0.033. For unknown peers, the corresponding values were *χ*^2^(17) = 35.708, *p* = 0.005, CFI = 0.994, TLI = 0.990, RMSEA = 0.046 (90% CI [0.024, 0.067]), and SRMR = 0.040. The analysis supported configural and first-order metric invariance, as well as equal item thresholds, across conditions. Second-order metric invariance was also supported: constraining the defending and inclusion loadings on the HCPB factor to equality resulted in ΔCFI = −0.002, ΔRMSEA = 0.012, and ΔSRMR = 0.004, all within commonly recommended criteria for measurement invariance. The latent correlation between HCPB toward known and unknown peers was 0.797 (95% CI [0.738, 0.856]), indicating a strong association while remaining below unity. In addition, the latent mean of HCPB toward unknown peers was significantly lower than that toward known peers (ΔM = −0.478, SE = 0.040, z = −12.014, *p* < 0.001). These findings support the comparability of the measure for assessing HCPB toward known and unknown school peers. The second-order HCPB factors showed low-to-moderate reliability, with ω = 0.602 for prosociality toward known peers and ω = 0.670 for prosociality toward unknown peers.

(c) Aggression scale (López & Orpinas, 2012). This instrument was produced and validated for use in Chile by López and Orpinas (2012). It is a self-report instrument that measures physical and verbal aggression among students through 11 Likert items and two factors (1–6 response format): (a) physical–verbal aggression and (b) anger. The Chilean version by López and Orpinas (2012) exhibited adequate reliability (*α* = 0.86) and appropriate fit indicators (*χ*^2^ = 183.6, *p* < 0.001; *CFI* > 0.90; *RMSEA* = 0.08). In the present study, McDonald’s ω was 0.918 for physical–verbal aggression, 0.791 for anger, and 0.929 for the total scale.
(d) Personal Well-Being Index for Adolescents. The scale was designed by Cummins et al. (2003) and adapted and validated for use in Chile by Alfaro et al. (2014). It is a one-factor self-report scale that measures overall well-being in adolescents through 7 Likert-type items. The scale uses a 1–10 response format, which was retained as originally developed by the authors. The version developed by Alfaro et al. (2014) exhibited acceptable reliability (*α* = 0.79) and goodness-of-fit (*χ*^2^ = 22.45, *p* < 0.02; *CFI* > 0.987; *TLI* = 0.976; *RMSEA* = 0.044). In the present study, McDonald’s ω for the seven-item well-being scale was 0.884.
(e) School Social Climate Questionnaire. It is a self-report scale composed of 16 Likert-type items that measure secondary school students’ perception of school climate on two dimensions: faculty social climate and school social climate. It was adapted and validated for use in Chile by Guerra et al. (2011), who reported that the factor analysis performed yielded adequate values (Kaiser–Meyer = 0.91; Bartlett’s test of sphericity = [*χ*^2^(91) = 4131.384; *p* < 0.01]). In the present study, McDonald’s ω was 0.854 for school-center climate, 0.803 for teacher climate, and 0.900 for the total scale.

### 2.4. Data Analysis

The data were analyzed using the R software environment version 4.6.0 (2026-04-24 ucrt) and AMOS. No imputation procedure was applied. For the descriptive analyses, correlations, and group comparisons, analyses were conducted using the available observations, resulting in different sample sizes across analyses. For the structural equation models, cases with missing data were excluded using listwise deletion, resulting in a complete-case sample of 475 participants. For the first specific objective, descriptive statistics were calculated for the variables studied. To compare HCPB levels by group, the Kruskal–Wallis and Mann–Whitney U tests were used. To compare HCPB toward known and unknown peers, the Wilcoxon signed-rank test for matched samples was used. For the second specific objective, the variables studied were correlated using Spearman’s rank correlation coefficient.

Finally, for the third objective, two structural equation models were tested. The first model examined the structural paths involving HCPB toward unknown peers, while the second model examined HCPB toward known peers. The test of univariate normality yielded skewness values ranging from −1.38 to 1.76 and kurtosis values from −0.91 to 2.52, which is within the acceptable range for maximum likelihood estimation (Kline, 2023). Consequently, and considering the size of the sample, maximum likelihood estimation was applied to the models. In addition, to increase the robustness of the estimations and the confidence intervals in the event of possible moderate deviations from normality, a procedure comprising 5000 resamples was implemented using bias-corrected bootstrap confidence intervals.

The next step consisted in specifying, identifying, estimating, and evaluating the structural models used to estimate the relationships with school climate based on HCPB, school aggression, and student well-being. Model fit was evaluated following the recommendations laid out by Kline (2023). Several values were used as a reference, being adopted as guidelines and not as absolute cutoff points (Kline, 2023): CMIN/df lower than 5, CFI and TLI higher than 0.90 (≥0.95 as excellent fit), RMSEA lower than 0.08 (≤0.06 as excellent fit), and IFI close to one.

## 3. Results

### 3.1. Descriptive Results of the Variables Studied

HCPB levels were close to the midpoint of the 1–5 scale (M = 3.35 for known peers; M = 3.05 for unknown peers), with inclusion showing higher mean values than defense in both groups (3.81 vs. 2.88; 3.57 vs. 2.53). School aggression was below the midpoint of the 1–6 scale (M = 1.90), whereas well-being was above the midpoint of the 1–10 scale (M = 7.29). Table 1 shows the descriptive statistics of the variables studied.

### 3.2. Costly Prosocial Behavior by Sex and Grade

Significant differences by sex were found in the overall measure of HCPB toward known students and in the dimension Inclusion toward known students, with small effect sizes and girls showing higher mean ranks than boys. Likewise, significant differences were observed in Inclusion toward unknown students, with a small effect size and, again, higher scores for girls. No statistically significant differences were found in the Defense dimensions or in HCPB toward unknown students. Table 2 lays out these results.

In addition, the Kruskal–Wallis test was used to examine differences between different measures of HCPB depending on the participants’ grade, which revealed statistically significant disparities in overall HCPB toward known peers (*H*(3) = 11.528, *p* = 0.009, ε^2^ = 0.016), indicating a small effect size. Mean ranks indicate that eleventh grade students reached the highest scores (*MR* = 301.32), followed by those in twelfth grade (*MR* = 280.35), tenth grade (*MR* = 266.89), and ninth grade (*MR* = 241.96). Bonferroni-corrected post-hoc comparisons only revealed a significant difference between ninth and eleventh grade students (adjusted *p* = 0.006), with the latter exhibiting higher HCPB levels than the former.

Significant differences were also found in the Defense dimension of HCPB toward known peers (*H*(3) = 11.581, *p* = 0.009, ε^2^ = 0.016), indicating a small effect size. Mean ranks indicated higher scores for eleventh grade students (*MR* = 295.74), followed by those in twelfth grade (*MR* = 294.83), tenth grade (*MR* = 266.74), and ninth grade (*MR* = 241.35). Bonferroni-corrected post-hoc comparisons only yielded a significant difference between ninth and eleventh grade students (adjusted *p* = 0.015), with the latter showing higher HCPB values than the former. The rest of the comparisons failed to reach statistical significance.

Finally, the Wilcoxon signed-rank test for matched samples was used to compare costly prosociality toward known and unknown students. The test revealed statistically significant differences between these conditions, *W* = 83,409, *Z* = 10.13, *p* < 0.001, with a moderate effect size (*r* = 0.443). Costly prosociality toward known peers exhibited higher scores (*Mdn* = 3.38) than that toward unknown peers (*Mdn* = 3.00).

### 3.3. Correlation of the Variables with HCPB Toward Unknown Students

Table 3 shows that costly prosocial behavior toward unknown peers was positively associated with well-being and school climate. With respect to the association with school aggression, some smaller-magnitude negative correlations were observed.

### 3.4. Correlation of the Variables with HCPB Toward Known Students

HCPB toward known students exhibited positive correlations with well-being and school climate. Likewise, negative associations were observed with most dimensions of school aggression and its overall measure (Table 4).

### 3.5. School Climate Predictive Models

Two structural equation models were evaluated to analyze the statistical relationships with school climate based on HCPB, well-being, and school aggression. Since there is prior evidence for the structural validity and internal consistency of the school climate and school aggression scales, school climate and school aggression were incorporated into the model as latent variables, with their respective dimensions specified as observed indicators. HCPB was incorporated into the model as a second-order latent factor, with two first-order latent factors, Defending and Inclusion, each indicated by four observed items. The models were identified by fixing the first loading of each latent factor to 1 and constraining the two second-order loadings of HCPB to equality. This was done to reduce the complexity of the model and the number of parameters to be estimated, thus increasing the parsimony and stability of the estimations, given that the main objective of the study was to evaluate structural relationships among variables rather than to re-examine the models used to measure said scales (Kline, 2023).

Model 1 incorporated HCPB toward unknown students, while Model 2 incorporated HCPB toward known students. Both models exhibited adequate fit indicators for most estimators. The modification indices made it possible to establish correlations between the errors of items 3 and 4, yielding re-specified versions of Models 1 and 2. This was due to their similarities in terms of semantic content and the fact that both refer to the same specific situation, defending, which may generate additional shared variance not explained by the latent factor evaluated. The re-specified models showed adequate fit indicators for all estimators.

Descriptively, Model 1 explained 34% of the school climate variance, whereas Model 2 explained 27%. In addition, HCPB toward unknown peers did not show a significant structural relationship with school aggression (β = −0.11, *p* = 0.073), whereas HCPB toward known peers showed a negative and significant structural relationship with this variable (β = −0.25, *p* < 0.05). In both models, well-being showed a positive structural relationship with school climate, whereas school aggression showed a negative structural relationship with it. Taken together, these models showed different patterns of associations between HCPB and the school variables according to the type of beneficiary. Table 5 shows these results, while Figure 1 and Figure 2 illustrate the models tested.

Bootstrap analyses were conducted to examine the direct, indirect, and total associations. For known peers, HCPB showed a significant direct negative relationship with school aggression (B = −0.296, 95% CI [−0.603, −0.018]). The indirect association of HCPB through aggression was significant for well-being (B = 0.152, 95% CI [0.036, 0.368]) and school climate (B = 0.057, 95% CI [0.009, 0.129]). The sequential indirect association through aggression and well-being was also significant (B = 0.021, 95% CI [0.005, 0.059]), whereas the indirect association through well-being alone was not (B = 0.042, 95% CI [−0.011, 0.096]). The total indirect association with school climate was significant (B = 0.120, 95% CI [0.053, 0.205]), as were the total associations with well-being (B = 0.456, 95% CI [0.110, 0.818]) and school climate (B = 0.464, 95% CI [0.313, 0.639]). For unknown peers, the direct relationship between HCPB and aggression was not significant (B = −0.082, 95% CI [−0.238, 0.016]), and none of the specific indirect associations was significant. However, the total indirect association with school climate was significant (B = 0.052, 95% CI [0.011, 0.102]), as were the total associations with well-being (B = 0.252, 95% CI [0.038, 0.491]) and school climate (B = 0.368, 95% CI [0.251, 0.484]).

## 4. Discussion

The general objective of this study was to examine the associations between costly prosociality toward known and unknown school peers and well-being, school aggression, and school climate among Chilean secondary school students by estimating structural paths from costly prosociality to these variables. Overall, an association was found between these variables consistent with prior scientific evidence (Li et al., 2023; Manzano-Sánchez et al., 2021; C. Wang et al., 2022). In addition, a novel finding is that the structural models yielded different patterns of structural relationships according to whether the beneficiary was known or unknown.

The first specific objective was to characterize HCPB levels in secondary school students, comparing them by sex, grade, and type of beneficiary (known/unknown). HCPB was hypothesized to differ by type of beneficiary, sex, and grade. With respect to sex, girls exhibited higher levels of costly prosociality in some of the dimensions evaluated.

Regarding prosocial behavior in general, the literature has generally found evidence of higher levels in women, which could be partly due to different ways of manifesting prosocial behaviors and to gender stereotypes (Nielson et al., 2017). In contrast, with respect to HCPB, it has been reported that men are more likely to exhibit risky and agentive helping behaviors, while women tend to engage in caregiving and interpersonal support (Hammond et al., 2023). In line with the results of this study, Duell and Steinberg (2020) found more positive risky behaviors in women than in men, including dangerous prosocial behaviors. However, other researchers have found no evidence of sex-based differences (Duell et al., 2025). In this regard, Duell et al. (2025) acknowledge that certain specific types of risky behavior could probably manifest themselves differentially when comparing by sex.

In consequence, HCPB appears to have a complex relationship with sex. The findings of this study suggest that this association may vary according to characteristics of the beneficiary, the type of HCPB, and the context where the help is rendered. The present study provides evidence of this association when the beneficiaries are known and unknown, when costly prosociality involves defending and including, and when these interactional dynamics take place in school settings.

With respect to educational level, overall HCPB and the dimension defense of known peers were found to be higher. In this regard, there is evidence that costly prosociality (e.g., defensive HCPB) increases during secondary education (Hammond & Brownell, 2015), especially toward one’s group (Armstrong-Carter & Telzer, 2025), and that it gradually decreases as one approaches adulthood (Padilla-Walker et al., 2018).

One possible explanation for the higher levels of HCPB observed in eleventh grade participants is that, during this period, students are consolidating their social bonds, which may be related to greater social connectedness and integration and may be associated with the manifestation of such behaviors (Armstrong-Carter et al., 2021; Hammond & Brownell, 2015). In contrast, the start of secondary education might be a stage of exploration, progressive development of social skills, and adaptation to the educational system (Rose et al., 2025), while during the final year—twelfth grade—students’ interests are likely to be focused on new job-related or educational demands (Mata-Correas et al., 2025). This makes it possible to hypothesize the existence of a midpoint of maximum psychosocial integration in educational trajectories, characterized by high levels of belonging, social integration, and commitment to others, which may be associated with the manifestation of HCPB. Future studies could be conducted to test this hypothesis.

Finally, in line with the available evidence, HCPB toward known students was found to be higher than that directed at unknown peers. In this regard, Waasdorp et al. (2022) have noted that, as they progress in secondary education, students gradually ascribe more value to friendship bonds, which may be linked to costly prosocial behaviors toward group members. To explain this phenomenon, Armstrong-Carter and Telzer (2025) examine the role of interpersonal closeness, empathy, and sense of belonging, which they regard as potential factors associated with HCPB toward known peers. On a practical level, these results could orient educational policies aimed at promoting active student participation in violence reduction and school climate improvement initiatives, bearing in mind that it would be necessary to plan and implement specialized interventions to promote HCPB toward different types of beneficiaries.

The second objective of the study was to determine how HCPB toward known and unknown peers correlated with well-being, school aggression, and school climate. A link between these variables was hypothesized, with the analyses performed revealing a positive association between HCPB toward both known and unknown peers, well-being, and school climate. In contrast, HCPB toward known students exhibited more negative correlations with school aggression and its dimensions than HCPB directed at unknown peers.

Some authors have reported evidence similar to that presented in this article, describing positive associations between costly prosociality, school climate (Freeman et al., 2024), and well-being (Tomar, 2023). With respect to school climate, in this study, HCPB toward both known and unknown peers was linked to the two dimensions of the instrument used: institutional climate, which measures perceived safety and positive relationships; and faculty climate, which measures the quality of the interpersonal relationships between students and teachers (Guerra et al., 2011). One possible explanation for this phenomenon is that defense and inclusion behaviors may be associated with positive responses in the beneficiaries such as gratitude, safety, a sense of belonging, and well-being, in addition to being related to friendship ties (Monks & Rix, 2024), thus being associated with a more positive school climate.

With respect to well-being, evidence for a link with prosociality is less consistent (Hirani et al., 2022; Tomar, 2023). This study provides new evidence in support of a positive association with well-being, with the correlation being slightly stronger when helping known peers. The limited consistency among the studies that have examined the relationship between costly prosociality and well-being may be due to the role of other variables, such as students’ emotional regulation capacity when providing help and their knowledge about how to help in these contexts (Cuadra et al., 2026), which may be associated with greater well-being.

The link found between HCPB and school aggression is a noteworthy finding, as more negative correlations were observed when costly prosociality is directed at known peers. Even though several studies that have examined general prosociality (Freeman et al., 2024) and defensive prosociality (Chicote-Beato et al., 2026; Willems et al., 2024) have reported a negative association with school violence, the present study suggests that the link between HCPB and school aggression may depend on the type of beneficiary.

Finally, in order to fulfill the last specific objective, two structural models of school climate were evaluated, considering HCPB, well-being, and school aggression. These models were specified to examine the structural paths from HCPB toward known and unknown peers to psychological well-being, school aggression, and school climate, as well as the structural paths from psychological well-being and school aggression to school climate. In the model involving unknown students, the path from HCPB to school aggression was not statistically significant, whereas it was statistically significant in the model involving known peers. The bootstrap analysis was consistent with this pattern: the confidence interval for the direct association between HCPB and school aggression included zero for unknown peers, whereas it excluded zero for known peers. Descriptively, the model involving unknown peers accounted for 34% of the variance in school climate, compared with 27% in the model involving known peers.

Regarding the above, the first aspect to consider is the role of the beneficiary. Although costly prosociality tends to benefit persons within the benefactor’s group (Armstrong-Carter & Telzer, 2025), the findings presented in this article indicate that, descriptively, the model involving HCPB toward unknown peers accounted for a larger proportion of variance in school climate than the model involving HCPB toward known peers. A possible interpretation of this phenomenon is that prosocial behaviors aimed at unknown peers may be associated with school climate through processes that extend beyond established in-group relationships, potentially encompassing the school community more broadly. Thus, said behaviors may be related to the collective norms of respect, support, and inclusion that configure school climate (Castro et al., 2026). A second possible mechanism is that prosocial actions toward unknown peers may reflect generalized norms of cooperation that extend beyond established peer relationships, potentially being associated with a broader sense of safety, support, and inclusion within the school community.

It is worth noting that, in the model involving unknown peers, the association between HCPB and school aggression was not statistically significant. This observation may be related to the fact that school aggression is a relational phenomenon whose meaning and dynamics depend on the existing bond between the assailant and the victim (Monks, 2023). In this context, helping a known classmate could be part of a relational pattern that is less compatible with aggression toward the same peer group. In contrast, when prosocial behavior is directed at an unknown peer, said behavior could represent a one-off episode that does not necessarily reflect the student’s usual prosocial interaction pattern, which may help explain the absence of a statistically significant association with school aggression. Future studies could focus on testing these hypotheses.

The above finding is especially relevant because the literature shows that supporting victims of school aggression is a protective factor against bullying (Chicote-Beato et al., 2026); however, the present findings suggest that the pattern of this relationship may vary according to the type of beneficiary.

The bootstrap analyses further showed different patterns of indirect associations according to beneficiary type. For known peers, the indirect association between HCPB and well-being through school aggression was statistically supported, as was the indirect association between HCPB and school climate through aggression. The sequential indirect association through aggression and well-being was also statistically supported, whereas the indirect association with school climate through well-being alone was not statistically supported. In contrast, none of the specific indirect associations was statistically supported in the model involving unknown peers. Nevertheless, the total indirect association between HCPB and school climate was statistically supported in both models.

Another point to consider is the positive structural relationship between HCPB and well-being. At present, the available evidence is contradictory and inconclusive regarding the relationship between these variables (Hirani et al., 2022; Tomar, 2023). In this study, the structural relationship between HCPB and student well-being was found to be positive, which is in line with research that has linked this type of prosociality with greater life satisfaction and well-being (Tomar, 2023). However, the bootstrap confidence intervals for the direct association between HCPB and well-being included zero in both models, which warrants caution in interpreting this relationship as a statistically supported direct association. The total association between HCPB and well-being was nevertheless statistically supported in both models. It is also worth noting that the present study only utilized an overall measure of well-being; therefore, it is not possible to specify which dimensions of student well-being are associated with HCPB.

The structural models generated highlight the potential relevance of beneficiary type when examining the structural relationships between HCPB and the school variables studied. This is an issue that educational policies on school climate and student witnesses of violence have not taken into account. Furthermore, these results suggest that the patterns of structural relationships between HCPB and the school variables may vary according to the relational context in which prosocial behavior occurs.

The models developed show that HCPB is structurally related to school variables concerning individual well-being, interpersonal school aggression, and students’ perceptions of school climate, which suggests that this type of prosociality is a relevant resource for understanding the functioning of educational communities.

The present study has limitations. First, the convenience sample and the inclusion of students from only two public schools in Chile limit the generalizability of the findings to other student populations and educational contexts. Second, self-report measures may be subject to response bias. Third, the cross-sectional design does not allow temporal precedence to be established and therefore limits causal inference. Comparisons between the structural models should also be considered exploratory, as their separate estimation does not allow statistical differences between corresponding parameters to be established. The residual correlations, supported by prior validation of the CPAC scale and item similarity, represent model re-specifications that require replication in independent samples. Fourth, the cultural specificity of the Chilean educational context may limit applicability to other contexts. Fifth, only two manifestations of costly prosociality were measured, limiting the findings to inclusion and defense behaviors. Sixth, the fixed order of the HCPB conditions may have introduced memory or consistency effects. Finally, only school aggression was assessed as a dimension of school violence.

In conclusion, this study provides evidence that contributes to understanding the associations between HCPB, well-being, school aggression, and school climate. The results presented reveal significant associations between HCPB and the school variables examined, as well as structural relationships within the models tested. The bootstrap analyses provided additional support for the direct association between HCPB and school aggression according to beneficiary type, while also indicating that the direct association between HCPB and well-being should be interpreted with caution. They also highlight the importance of considering the patterns of structural relationships between HCPB and the school variables according to the type of beneficiary, which may be important for educational policies that encourage students to play an active role in tackling school violence and school climate issues. Taken together, the findings suggest that costly prosociality is not only an individual student characteristic, but a social resource associated with personal well-being and school climate quality. Descriptively, the model involving behaviors directed at more distant peers accounted for a larger proportion of variance in school climate, suggesting a potential new line of research on the role of the beneficiary of costly prosocial behavior in the functioning of educational communities.

## Figures and Tables

**Figure 1:**
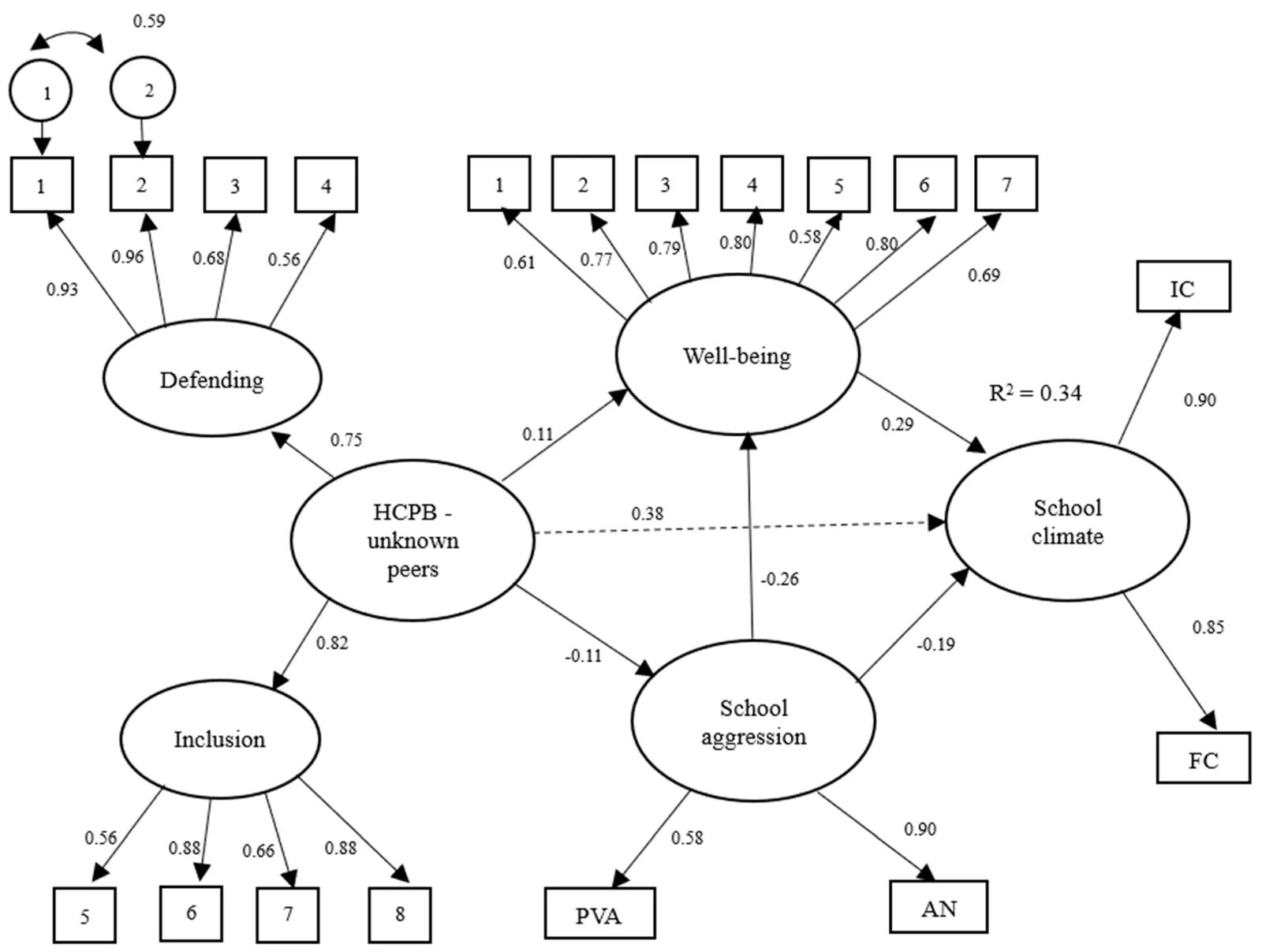
Model 1: Structural relationships between HCPB toward unknown peers and school climate. *Note*. The structural relationship between HCPB and school aggression was not significant (*p* = 0.073). The remaining structural relationships were statistically significant, with *p* values ranging from 0.001 to 0.046. IC = institutional social climate; FC = faculty climate; ANG = anger; PVA = physical and verbal aggression. The dashed arrow is used to prevent arrows from overlapping.

**Figure 2:**
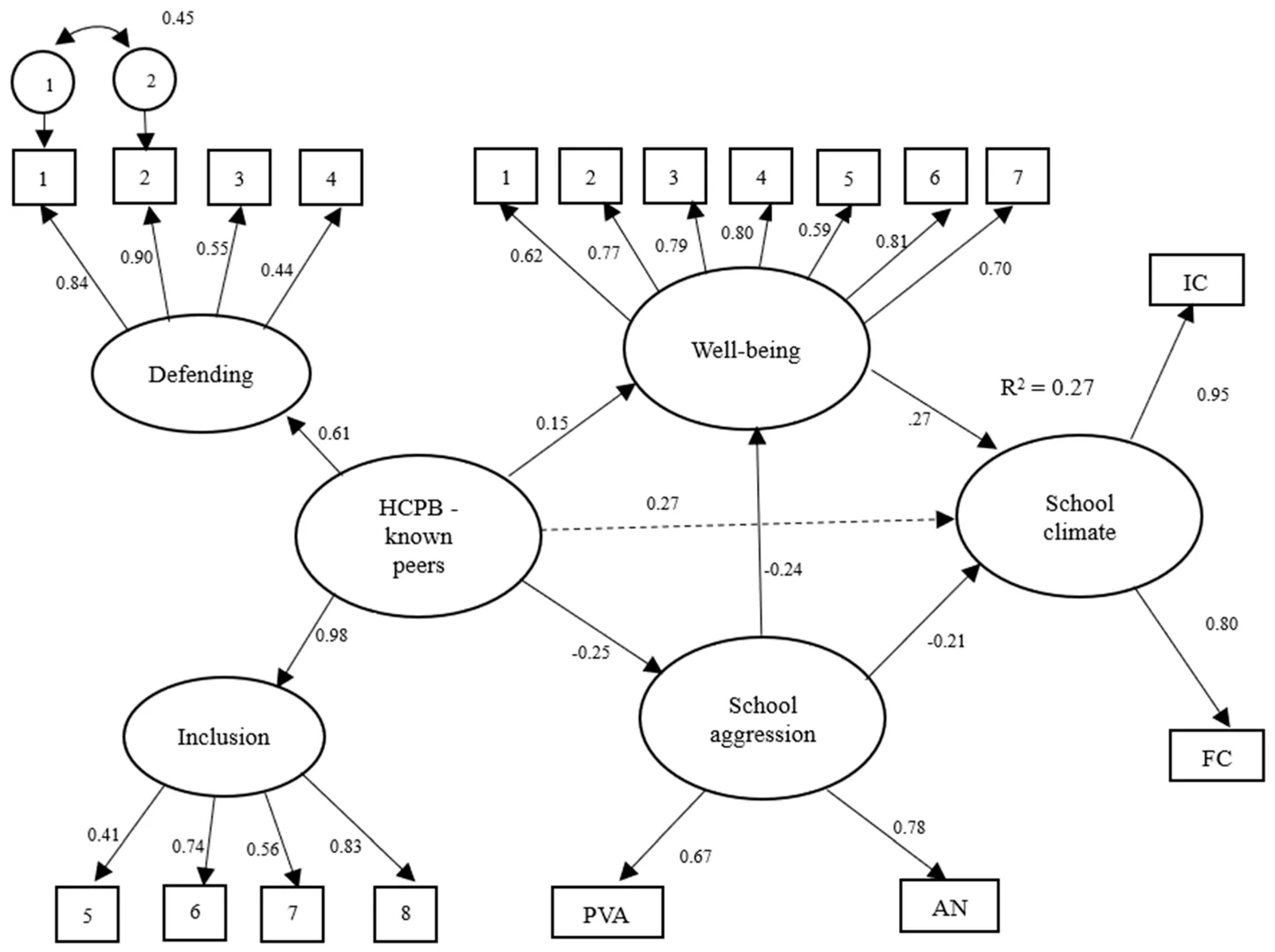
Model 2: Structural relationships between HCPB toward known peers and school climate. Note. All structural relationships were statistically significant, with *p* values ranging from 0.001 to 0.026. The dashed arrow is used to prevent arrows from overlapping.

**Table 1:** Descriptive statistics.

	N	Minimum	Maximum	Mean	*SD*
HCPB—known peers	529	1.00	5.00	3.35	0.755
Defense—known peers	529	1.00	5.00	2.88	0.932
Inclusion—known peers	529	1.00	5.00	3.81	0.859
HCPB—unknown peers	520	1.00	5.00	3.05	0.938
Defense—unknown peers	522	1.00	5.00	2.53	1.09
Inclusion—unknown peers	526	1.00	5.00	3.57	1.06
Well-being	521	1.00	10.0	7.29	1.99
School aggression	514	1.00	5.64	1.90	1.04
Verbal–physical school aggression	519	1.00	5.67	1.78	1.05
School aggression—anger	521	1.00	6.00	2.50	1.62
School Climate	502	1.00	5.00	3.23	0.732

**Table 2:** Costly prosocial behavior by sex.

Variable		*n*	Mean Rank	*U* Value	*Z* Value	*p* Value	*rrb*
HCPB—known peers				30,047.0	−2.640	0.008	0.133
	Boys	252	245.73				
	Girls	275	280.74				
Defense—known peers				33,944.0	−0.406	0.685	-
	Boys	252	261.20				
	Girls	275	266.57				
Inclusion—known peers				27,616.0	−4.047	<0.001	0.203
	Boys	252	236.09				
	Girls	275	289.58				
HCPB—unknown peers				30,297.0	−1.851	0.064	-
	Boys	245	246.66				
	Girls	273	271.02				
Defense—unknown peers				33,993.0	0.171	0.864	-
	Boys	246	261.68				
	Girls	274	259.44				
Inclusion—unknown peers				28,441.0	−3.375	0.001	0.171
	Boys	251	239.31				
	Girls	273	283.82				

*Note*. rrb = rank-biserial correlation (effect size).

**Table 3:** Correlation of the variables with HCPB toward unknown students.

	2	3	4	5	6	7	8	9	10
1. HCPB—unknown peers	0.868 **	0.843 **	0.156 **	−0.073	−0.033	−0.086	0.345 **	0.313 **	0.342 **
2. Defense—unknown peers	1.000	0.482 **	0.130 **	−0.004	0.075	−0.090 *	0.280 **	0.220 **	0.328 **
3. Inclusion—unknown peers		1.000	0.131 **	−0.135 **	−0.143 **	−0.074	0.322 **	0.332 **	0.267 **
4. Well-being			1.000	−0.174 **	−0.101 *	−0.239 **	0.348 **	0.344 **	0.307 **
5. School aggression				1.000	0.917 **	0.772 **	−0.287 **	−0.291 **	−0.208 **
6. Aggression—PV					1.000	0.506 **	−0.245 **	−0.263 **	−0.156 **
7. Anger						1.000	−0.257 **	−0.238 **	−0.219 **
8. School climate							1.000	0.943 **	0.917 **
9. Institutional social climate								1.000	0.739 **
10. Faculty climate									1.000

*Note*. * significant correlation at 0.05; ** significant correlation at 0.01; PV = physical and verbal. Correlations were calculated using pairwise available observations. Sample sizes ranged from 502 to 529 across correlations.

**Table 4:** Correlation of the variables with HCPB toward known students.

	2	3	4	5	6	7	8	9	10
1. HCPB—known peers	0.846 **	0.807 **	0.236 **	−0.155 **	−0.133 **	−0.141 **	0.302 **	0.306 **	0.256 **
2. Defense—known peers	1.000	0.393 **	0.206 **	−0.055	−0.009	−0.098 *	0.203 **	0.187 **	0.188 **
3. Inclusion—known peers		1.000	0.197 **	−0.211 **	−0.219 **	−0.136 **	0.311 **	0.334 **	0.243 **
4. Well-being			1.000	−0.174 **	−0.101 *	−0.239 **	0.348 **	0.344 **	0.307 **
5. School aggression				1.000	0.917 **	0.772 **	−0.287 **	−0.291 **	−0.208 **
6. Aggression—PV					1.000	0.506 **	−0.245 **	−0.263 **	−0.156 **
7. Anger						1.000	−0.257 **	−0.238 **	−0.219 **
8. School climate							1.000	0.943 **	0.917 **
9. Institutional social climate								1.000	0.739 **
10. Faculty climate									1.000

*Note*. * significant correlation at 0.05; ** significant correlation at 0.01; PV = physical and verbal. Correlations were calculated using pairwise available observations. Sample sizes ranged from 502 to 529 across correlations.

**Table 5:** Fit indicators of the structural models evaluated.

Model	*χ* ^2^	df	*χ*^2^/df	*p*	CFI	TLI	IFI	RMSEA	IC90_inf	IC90_sup
Model 1: HCPB—unknown peers	628.09	144	4.36	<0.001	0.901	0.883	0.902	0.084	0.078	0.091
Model 1: HCPB—unknown peers, re-specified	435.25	143	3.04	<0.001	0.940	0.929	0.941	0.066	0.059	0.073
Model 2: HCPB—known peers	489.365	144	3.39	<0.001	0.910	0.893	0.911	0.071	0.064	0.078
Model 2: HCPB—known peers, re-specified	387.405	143	2.71	<0.001	0.936	0.924	0.937	0.060	0.053	0.067

## Data Availability

The data supporting the findings of this study are not publicly available due to ethical and confidentiality considerations. According to the informed consent and assent obtained from the participants, the research data are confidential and may only be accessed and managed by the principal investigator and the research team. The data are securely stored and will be retained for a maximum of five years to allow for potential reanalyses within the framework of the research. Therefore, the dataset cannot be made publicly available.
